# A Study on Gallbladder Carcinoma With Special Reference to Risk Factors

**DOI:** 10.7759/cureus.103570

**Published:** 2026-02-13

**Authors:** Sthapana Sharma, Ashfia Habib, Deepjyoti Das, Bhabesh Kumar Das, Jutika Ojah

**Affiliations:** 1 Department of Community Medicine, Gauhati Medical College and Hospital, Guwahati, IND; 2 Department of Surgical Oncology, State Cancer Institute, Guwahati, IND

**Keywords:** assam, gallbladder cancer, hospital-based study, lifestyle, metabolic syndrome, north-east india, risk factors

## Abstract

Introduction: Gallbladder cancer (GBC) has been identified as one of the most fatal forms of gastrointestinal cancers worldwide by exhibiting a high incidence in North-Eastern India. This cancer mostly presents at an advanced stage, and various lifestyle, metabolic, and environmental elements have been linked to the onset. The objectives of the study were to evaluate the risk factors for gallbladder carcinoma and to establish the incidence pattern among patients visiting a tertiary cancer centre in the state of Assam.

Methods: A hospital-based prospective study was conducted for six months, starting from May to October 2024, at the State Cancer Institute, Guwahati. Patients diagnosed with carcinoma of the gallbladder and attending the Surgical Oncology Outpatient Department were included in the study after obtaining informed consent on the day of data collection. Data were collected using a pre-tested structured proforma. Data analysis and visualisations for this study were assisted using *Julius AI* (Rahul Sonwalkar, San Francisco, CA, USA). Results are presented using descriptive and inferential statistics, including chi-square tests.

Results: The study included 25 patients, with a mean age of 56.0 ± 14.7 years. Most participants were classified as middle-aged or elderly. Males constituted 16 (64%) of the cases, and 15 (60%) patients resided in rural areas. The mean BMI was 25.3 kg/m². Hypertension was present in 13 (52%) patients and diabetes mellitus in 10 (40%). Alcohol consumption was reported by 15 (60%), while 6 (24%) were current smokers. A non-vegetarian diet was followed by 16 (64%) of the participants, and 8 (32%) reported a family history of cancer. No statistically significant associations were observed between smoking and alcohol use (*p* = 0.255), hypertension and diabetes (*p* = 1.000), or BMI and hypertension (*p* = 0.825). However, there was clear clustering of modifiable risk factors among patients. The continued caseload at the centre reflected the persistent high regional burden of GBC.

Conclusions: Gallbladder carcinoma is recognised to remain a serious health problem in the state of Assam, mostly prevalent amongst the elderly population and in rural areas. The high incidence of exposures to metabolic and lifestyle factors strongly underscores the multifactorial theory of gallbladder carcinogenesis. Even though the statistical association had limitations due to sample size, the results strongly underscore the importance of targeted prevention and larger studies in high-incidence areas.

## Introduction

Cancer remains a significant global health challenge, responsible for nearly 10 million deaths in 2020. Factors such as tobacco use, high body mass index (BMI), alcohol consumption, inadequate fruit and vegetable intake, and lack of physical activity contribute to about one-third of these deaths. Infections like HPV and hepatitis, which can lead to cancer, are responsible for approximately 30% of cancer cases in low- and lower-middle-income countries [1]. According to the Global Cancer Observatory (GLOBOCAN), there were 19.3 million new cancer cases worldwide in 2020, with India ranking third after China and the United States. GLOBOCON forecasts that by 2040, cancer cases in India will increase to 2.08 million, representing a 57.5% rise from 2020 [2]. The Indian Council of Medical Research - National Centre for Disease Informatics and Research (ICMR-NCDIR) National Cancer Registry Programme projects a 12% increase in cancer cases in India by 2025 [3]. In 2017, the World Health Assembly passed a resolution on "cancer prevention and control" within an integrated framework, urging governments and the WHO to accelerate efforts to achieve the goals set in the Global Action Plan for the prevention and control of non-communicable diseases (2013-2020) and the 2030 UN Agenda for Sustainable Development Goals (SDGs) to reduce premature cancer mortality [4].

Since the establishment of the earliest cancer registries in 2003, the northeastern states of India have consistently reported high cancer incidence rates [5]. Among various cancers, gallbladder cancer (GBC) is more prevalent in females (3.7%) than in males (2.2%) compared to other gastrointestinal sites [6]. Furthermore, GBC is more commonly diagnosed in younger women aged 45 to 49 than other cancers [6]. GBC ranks among the deadliest gastrointestinal malignancies, with a poor overall survival. Key characteristics of this cancer include (a) a non-specific onset and symptomless progression, leading to late-stage detection and a poor prognosis, and (b) a distinct geographical pattern, with cases concentrated in river basin areas like the Ganga-Meghna-Brahmaputra (GMB) plain [7].

The Indian Council of Medical Research (ICMR) has identified various risk factors for GBC, including ethnicity, gender, age, gallstones, chronic inflammation, genetic predisposition, gallbladder polyps, and lifestyle choices [8]. The ICMR suggests that controlling obesity and diabetes, maintaining a diet rich in fruits and vegetables, and regular exercise can help prevent GBC [8]. GBC is regarded as a disorder with multiple contributing factors. Gallstones are considered the most significant risk factor. Only 1-3% of individuals with cholelithiasis develop GBC [9], and it is reported that 70-90% of Indian GBC patients have gallstones [10]. Despite this, region-specific data on the clinical and risk factor profile of GBC in Northeast India remain limited.

In this context, the present study was undertaken to evaluate selected metabolic and lifestyle-related risk factors associated with gallbladder carcinoma and to describe the pattern of presentation among patients attending a tertiary cancer care centre in Assam.

## Materials and methods

This hospital-based prospective study was conducted over a six-month period, from May 2024 to October 2024, at the State Cancer Institute, Guwahati, a tertiary-level cancer care centre. All eligible and diagnosed patients with carcinoma of the gallbladder attending the Surgical Oncology Outpatient Department (OPD) on the days of data collection and who provided written informed consent were included in the study. Recruitment was limited to the investigator’s scheduled data-collection visits.

**Figure 1:**
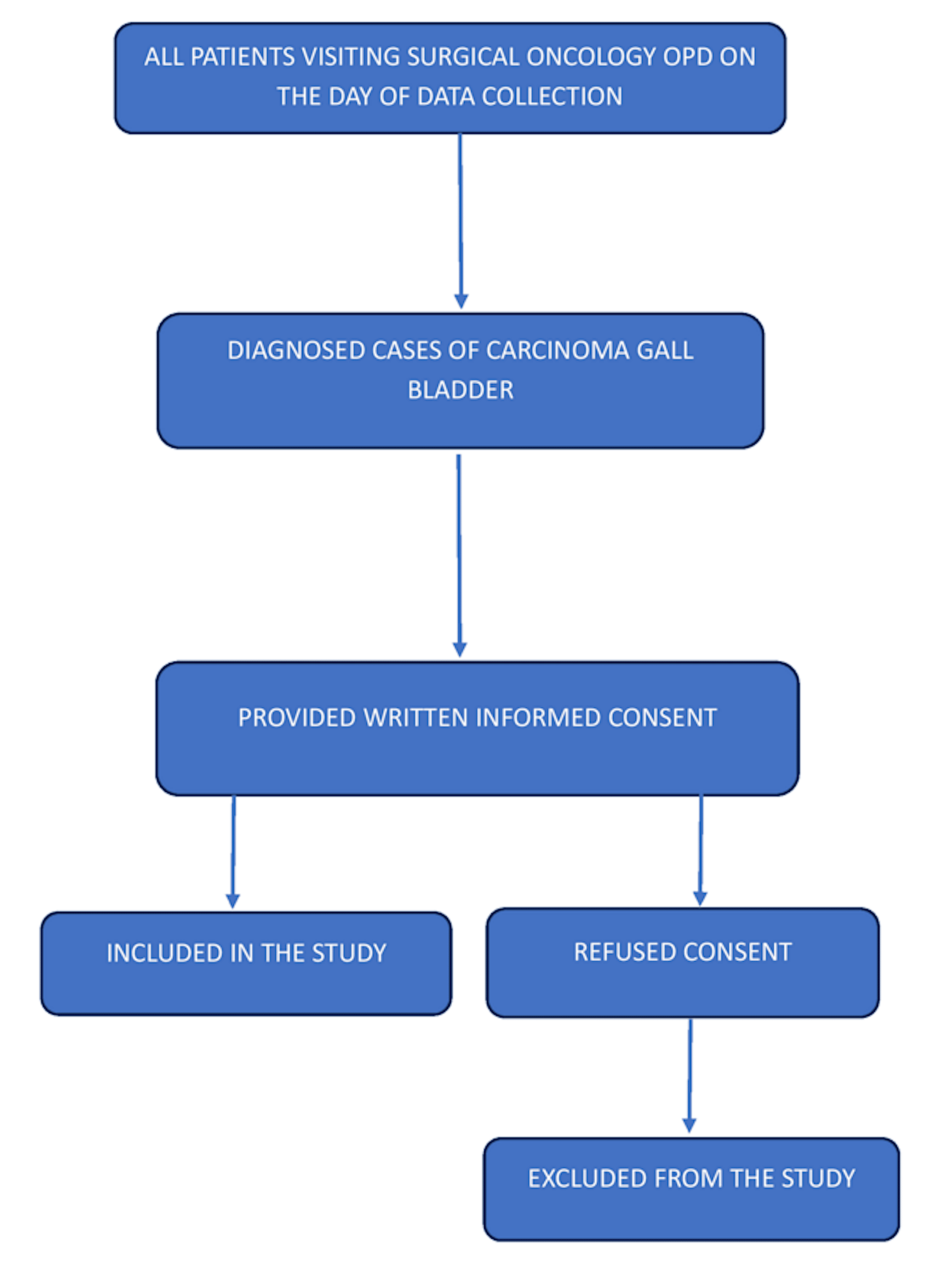
Process of patient recruitment

A total of 25 patients were enrolled during the study period, representing the complete accessible population of GBC cases available during those visits. Figure 1 depicts a flowchart for the patient recruitment process.

A non-probability consecutive sampling technique was employed. Data were collected using a pre-tested structured proforma through face-to-face interviews with the patients. All cases of GBC were classified according to the International Classification of Diseases for Oncology, Third Edition (ICD-O-3), using the topography code C23.9 for gallbladder and appropriate morphology codes to define histological type and malignant behaviour. All cases of GBC included in the study had a confirmed histopathological diagnosis. ICD-O-3 codes were assigned based on pathological reports available in the medical records. Detailed histological subtyping was not analysed, as it was beyond the scope of the present study. This standardised coding ensured uniformity and comparability with national and international cancer registry data. All participants provided written informed consent prior to enrolment, and confidentiality of the collected information was maintained.

A pre-tested structured proforma was used to collect information on participants’ sociodemographic characteristics, including age, sex, and place of residence. Data were also collected on anthropometric measurements, including BMI, metabolic comorbidities such as hypertension and diabetes mellitus, and behavioural and lifestyle factors, including tobacco use, alcohol consumption, and dietary patterns (vegetarian or non-vegetarian). In addition, information on the family history of cancer was recorded. All these variables were analysed and are presented in the results section.

Statistical analysis followed a predefined workflow involving data entry, descriptive summarisation, and bivariate analysis using the chi-square test as an exploratory approach to assess the co-occurrence of selected behavioural and metabolic risk factors among patients with gallbladder carcinoma, rather than to infer causality or to make clinical predictions. Data were analysed using Julius AI (Rahul Sonwalkar, San Francisco, CA, USA), an artificial intelligence-based data analysis platform, with the final outputs verified and approved by the investigators.

Ethical clearance was obtained from the Institutional Ethics Committee of the State Cancer Institute, Gauhati Medical College, Guwahati (approval no. SCI/GMC/ECR/2020/155) before commencement of the study.

## Results

**Figure 2:**
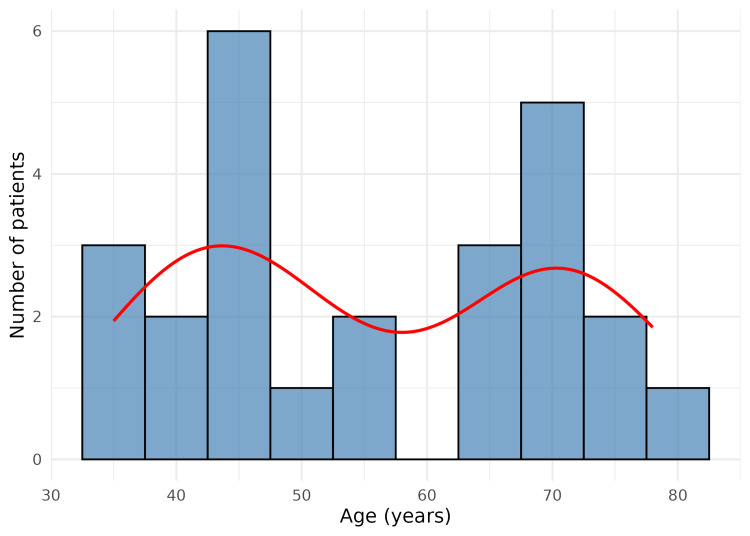
Age-wise distribution of gallbladder cancer patients This figure depicts the age distribution of patients with gallbladder carcinoma, presented as a histogram with an overlaid smoothed density curve. The x-axis indicates age in years, while the y-axis represents the number of patients, highlighting the overall age range and the concentration of cases within specific age groups.

This hospital-based prospective study included a total of 25 diagnosed cases of GBC. The mean age of the participants was 56.0 ± 14.7 years (range: 35-78 years), indicating that the disease predominantly affected middle-aged and elderly individuals. Figure 2 presents the age distribution of the study population.

Sixteen patients (64%) were male, and nine (36%) were female. A greater proportion of patients, 15 (60%), belonged to rural areas, while 10 (40%) were from urban areas.

**Figure 3:**
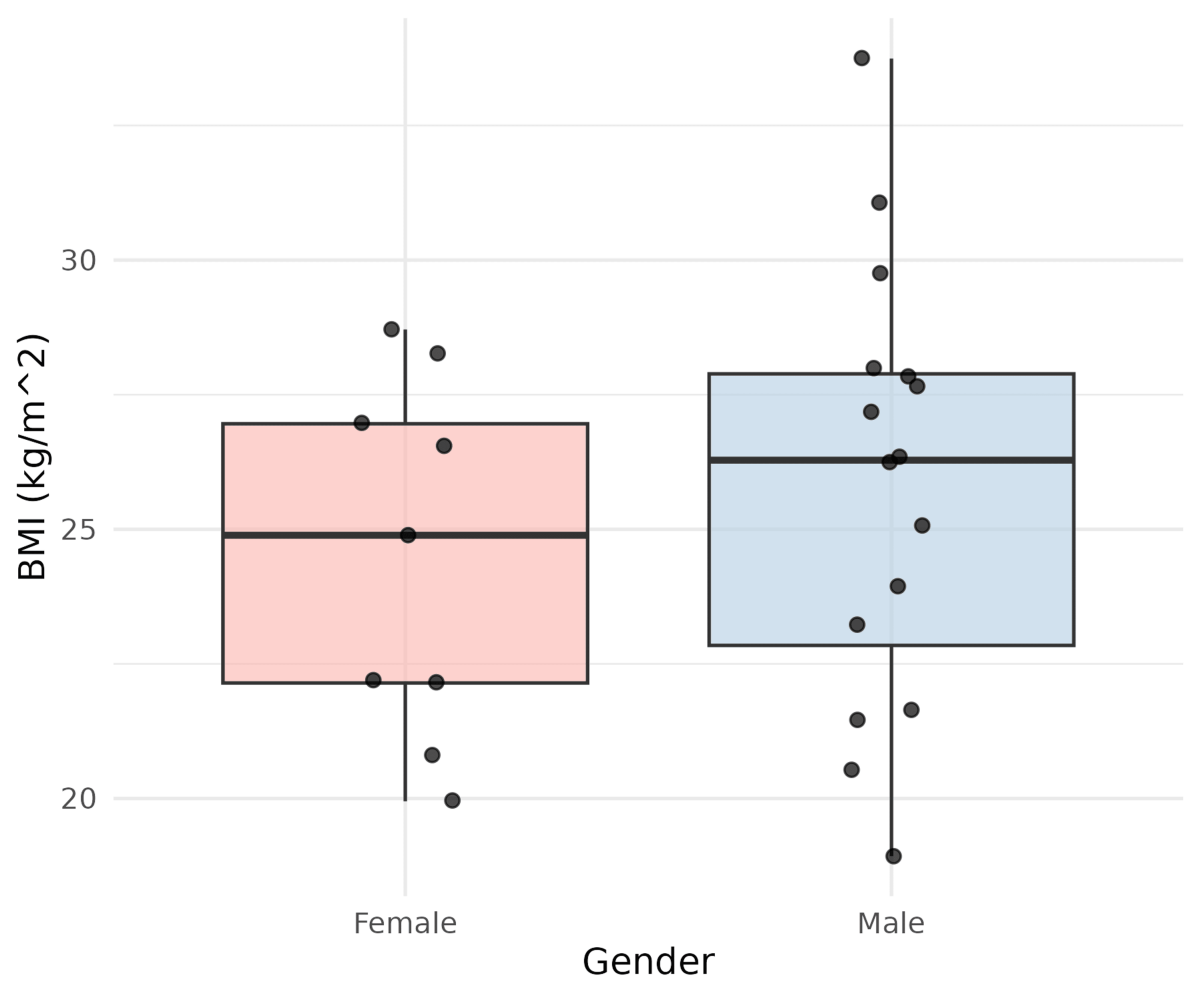
Gender-wise distribution of body mass index (BMI) among patients with gallbladder carcinoma This figure presents the distribution of BMI according to gender using boxplots with overlaid individual data points. The x-axis represents gender, and the y-axis shows BMI in kg/m². The plot allows for a visual comparison of the central tendency, spread, and outliers of BMI between male and female patients.

**Figure 4:**
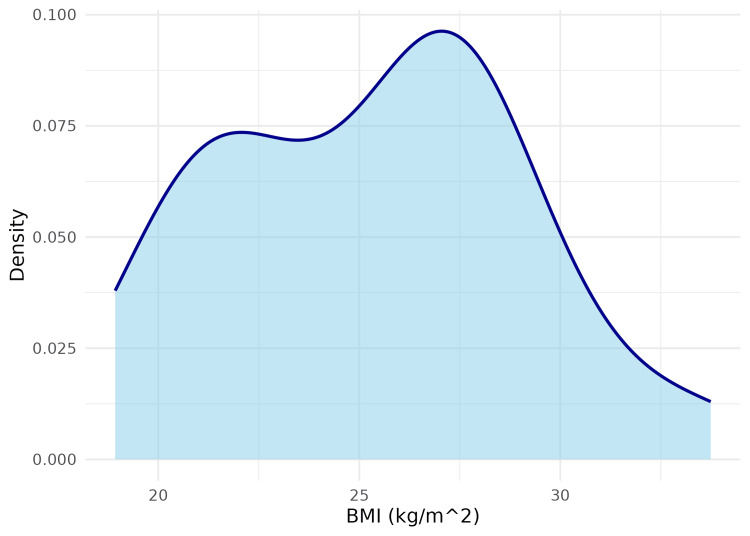
Smoothed density plot illustrating the distribution of body mass index (BMI) among the study participants This figure shows a smoothed probability density plot of BMI among the study participants. The x-axis represents BMI in kg/m², and the y-axis indicates the estimated density, demonstrating the overall shape of the BMI distribution and whether values tend to cluster within particular BMI ranges.

The mean BMI of the study participants was 25.3 kg/m², indicating an overall tendency towards overweight. The gender-wise distribution of BMI is shown in Figure 3, and the overall BMI density distribution is illustrated in Figure 4.

**Figure 5:**
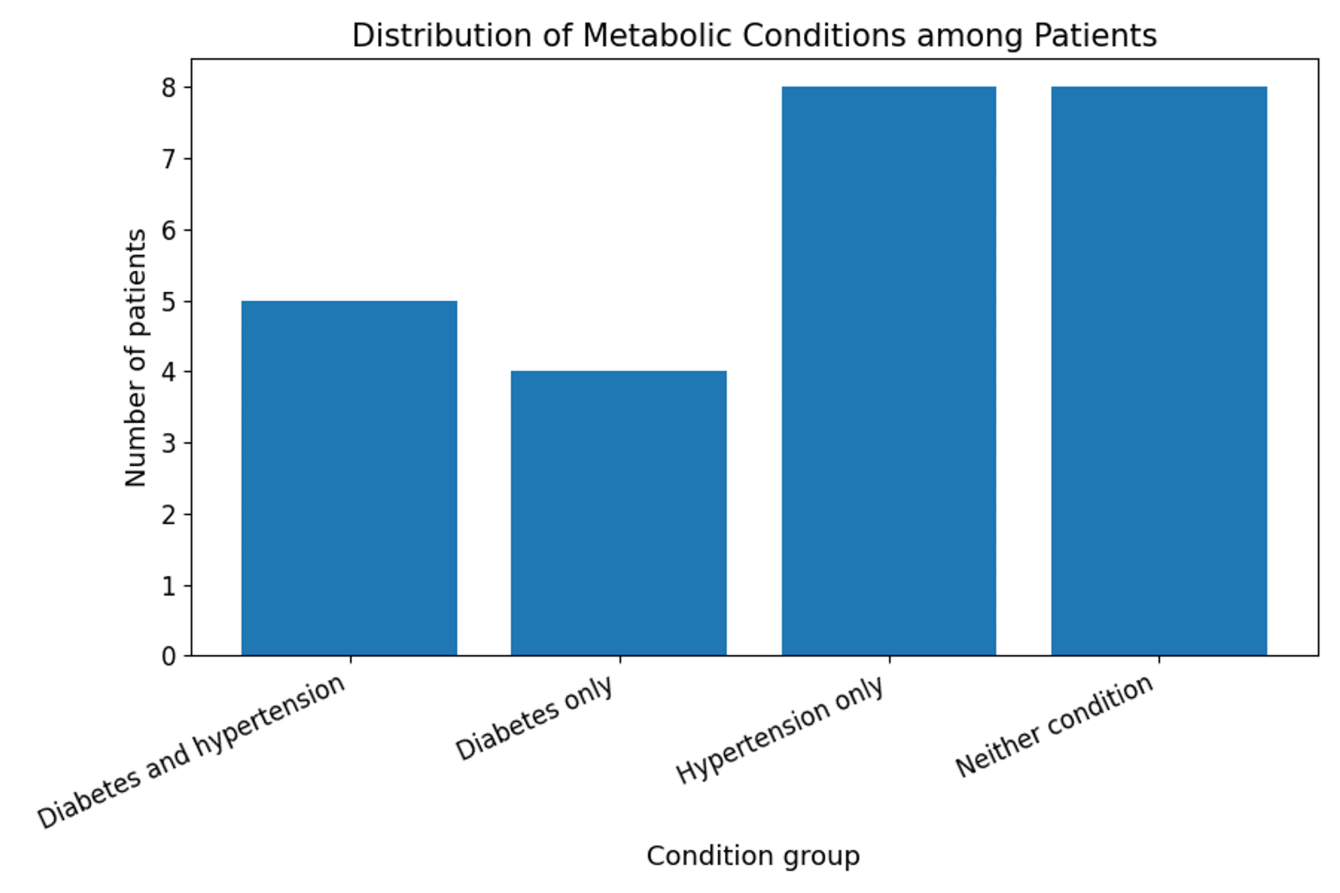
Co-existence of hypertension and diabetes mellitus among patients with gallbladder carcinoma This figure displays the co-existence of hypertension and diabetes among patients with gallbladder carcinoma. Patients are grouped into four categories on the x-axis: both diabetes and hypertension, diabetes only, hypertension only, and neither condition. The bar heights indicate the number of patients in each group, summarising the burden of these common comorbidities.

**Figure 6:**
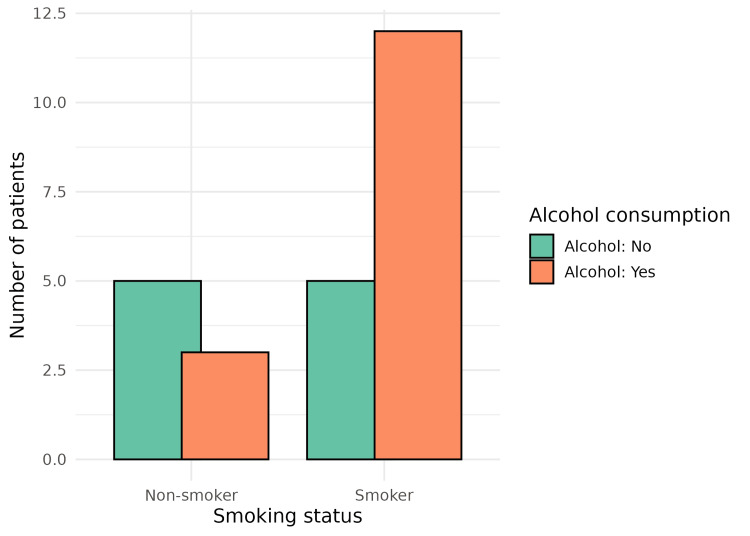
Distribution of alcohol consumption according to smoking status among study participants This figure illustrates the relationship between smoking status and alcohol consumption among the study participants. The bar chart compares the number of smokers and non-smokers on the x-axis, stratified by whether they consume alcohol, thereby showing how alcohol use co-occurs with tobacco smoking in this cohort.

A history of hypertension was present in 13 (52%) patients, while 10 (40%) were known cases of diabetes mellitus. Regarding behavioural and familial risk factors, 6 (24%) patients were current smokers, 15 (60%) reported alcohol consumption, 16 (64%) followed a non-vegetarian diet, and 8 (32%) had a positive family history of cancer. The co-existence of hypertension and diabetes is shown in Figure 5, and the overlap between smoking and alcohol consumption is depicted in Figure 6.

Bivariate associations between behavioural and metabolic risk factors were evaluated using the chi-square test. No statistically significant association was observed between smoking and alcohol consumption (χ² = 1.29, p = 0.255). Similarly, hypertension and diabetes mellitus did not show a significant association (χ² = 0.00, p = 1.000), and BMI category was not significantly associated with hypertension (χ² = 0.38, df = 2, p = 0.825), as shown in Table 1. Although these associations were not statistically significant, the findings should be interpreted in the context of the small sample size, which limits statistical power. Nevertheless, the co-occurrence of multiple metabolic and behavioural risk factors among patients remains epidemiologically relevant.

**Table 1:** Bivariate association between behavioural and metabolic risk factors among gallbladder carcinoma patients The table presents chi-square test results assessing the association between selected behavioural and metabolic risk factors among patients with gallbladder carcinoma. P-values < 0.05 were considered statistically significant. df = degrees of freedom; BMI: body mass index

Variable comparison	Chi-square value	df	p-value
Smoking vs. alcohol	1.29	1	0.255
Hypertension vs. diabetes	0.00	1	1.000
BMI category vs. hypertension	0.38	2	0.825

The continued presentation of GBC cases at this tertiary cancer referral centre during the study period highlights the persistent clinical burden of the disease in Assam, consistent with cancer registry reports indicating a high regional incidence in Northeast India.

## Discussion

The present study assessed the clinical profile and potential risk factors among patients with GBC attending a tertiary cancer centre in Assam, a region known to have one of the highest burdens of GBC in India. The disease predominantly affected middle-aged and elderly individuals, as reflected by the mean age of 56 years in our study population. This finding is consistent with the observations of Pandit et al., who reported a mean age at diagnosis of 52.5 years and a female predominance (67%) [11].

Gallstones are widely recognised as a major risk factor for GBC, and this was also observed in our study, in agreement with the findings of Huang et al. [12]. Kumar et al. further demonstrated marked geographic variation in the incidence of GBC in India, with significantly higher rates in the northern and northeastern regions compared to the southern states [13], which is in line with the regional pattern observed in our study. Our findings are also comparable to those of Mhatre et al., who reported an association between mustard oil consumption and an increased risk of GBC [14]. Similarly, Mishra et al., in a gallstone-matched analysis, identified several factors associated with GBC, including age ≥50 years, hypertension, and the use of antihypertensive medications [8], which parallels the metabolic comorbidity profile observed in our cohort.

Obesity is an important global public health concern and is known to be associated with metabolic disorders, gallstone disease, and various cancers. In the present study, the mean BMI of participants fell within the overweight range, and metabolic conditions such as diabetes and hypertension were commonly observed. These findings are consistent with those reported by Tan et al., who demonstrated an increased risk of GBC with obesity, particularly among women [15]. Although none of the bivariate associations examined in this study reached statistical significance, they help illustrate the tendency of several modifiable risk factors, such as obesity, hypertension, diabetes, tobacco use, and alcohol consumption, to occur together among patients with GBC. This clustering of risk exposures reflects the multifactorial nature of gallbladder carcinogenesis and highlights the need for integrated preventive and lifestyle-based strategies in populations at higher risk.

A key strength of this study is its focus on patients from Assam, a region with a disproportionately high burden of GBC compared with many other parts of India. The occurrence of the disease in middle-aged and younger patients further highlights this distinct regional pattern and suggests the possible role of region-specific environmental, lifestyle, or metabolic factors.

Our study has some limitations. The small sample size of 25 patients, resulting from recruitment restricted to OPD visits during the data collection period, may have limited the statistical power to detect significant associations. As this was a hospital-based study conducted at a tertiary care centre, the findings may not be generalisable to the wider population. The use of consecutive non-probability sampling may have introduced selection bias, and self-reported information on behavioural factors could be affected by recall bias. In addition, the cross-sectional design does not allow causal relationships to be established. Information on several established and suspected risk factors for GBC was not collected, including occupational exposure to industrial carcinogens, aflatoxin exposure, use of medications such as methyldopa, hormonal therapies, or isoniazid, as well as the presence or absence of gallstones, gallbladder polyps, or primary sclerosing cholangitis. As a result, a more comprehensive assessment of potential aetiological contributors, particularly among younger patients from this high-incidence region, was not possible. Future studies with larger sample sizes and more detailed assessments of environmental, occupational, and clinical exposures are needed to better understand these associations. Despite these limitations, the study provides important region-specific insights into GBC in a high-burden setting.

## Conclusions

Carcinoma of the gallbladder continues to be a major health issue in Assam, with the majority of patients falling into the elderly age groups. The presence of a high frequency of modifiable risk factors like obesity, hypertension, diabetes mellitus, alcohol consumption, and tobacco use strongly emphasises the complex aetiology of GBC. Although it was not possible to demonstrate statistical significance due to the small sample size, the co-existence of the risk factors underscores the importance of preventive measures and lifestyle changes in endemic areas.
